# Supplementary material for: Nutritional Characterization of Chilean Landraces of Common Bean
Source: Plants (Basel). 2024 Mar 12;13(6):817. doi: 10.3390/plants13060817 (PMC10974410; doi:10.3390/plants13060817)
Supplement: Supplementary file 1 [file plants-13-00817-s001.zip › supplementary.pdf]

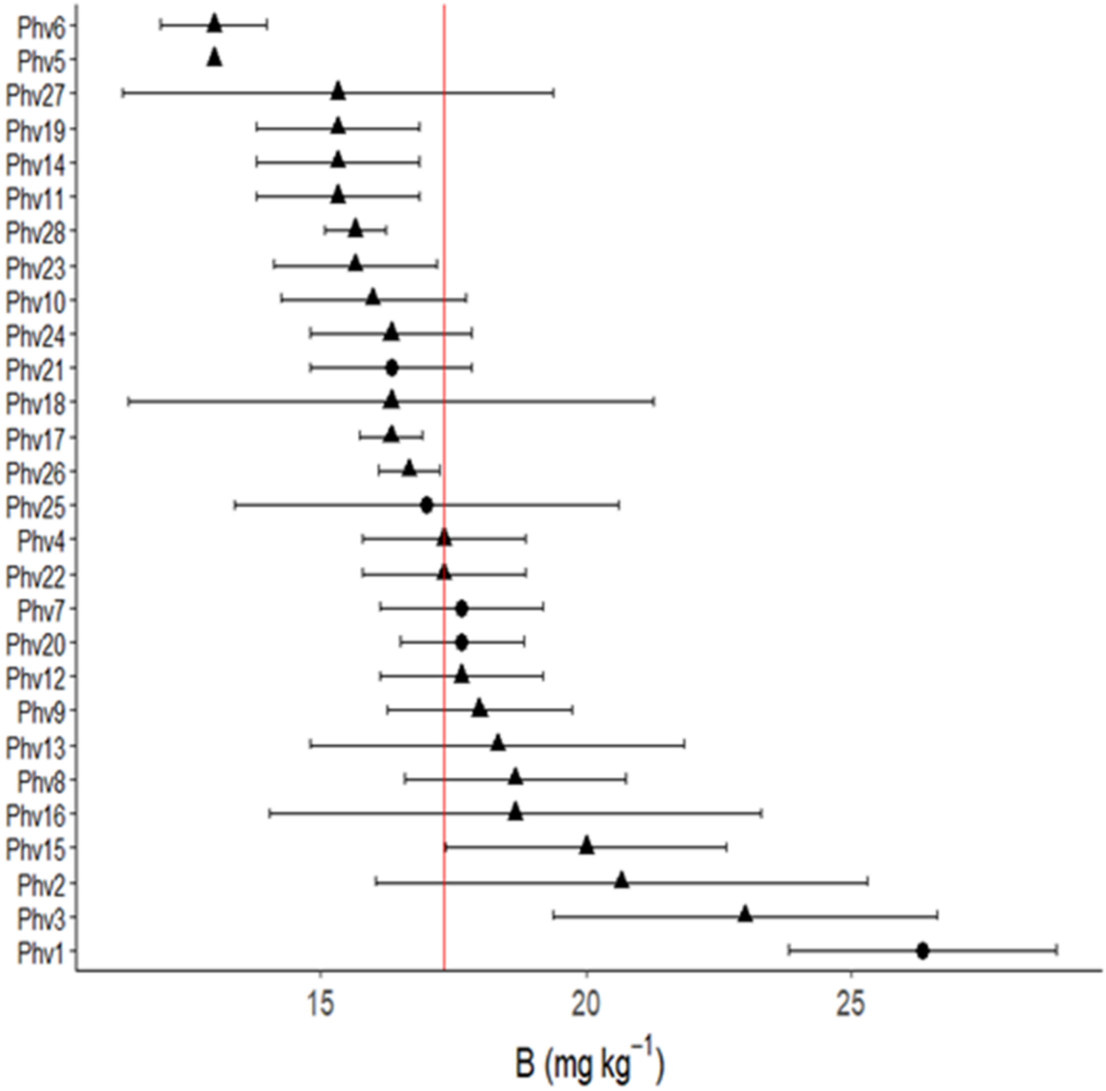

Figure S1. Concentration of Boron (B) for the 28 common bean accessions. Chilean landraces and commercial varieties are indicated with a triangle and a circle in the scatterplots, respectively. The red vertical line indicates the average boron concentration in all accessions. Significant differences between each accession according to the Tukey test are shown in the Table S2.

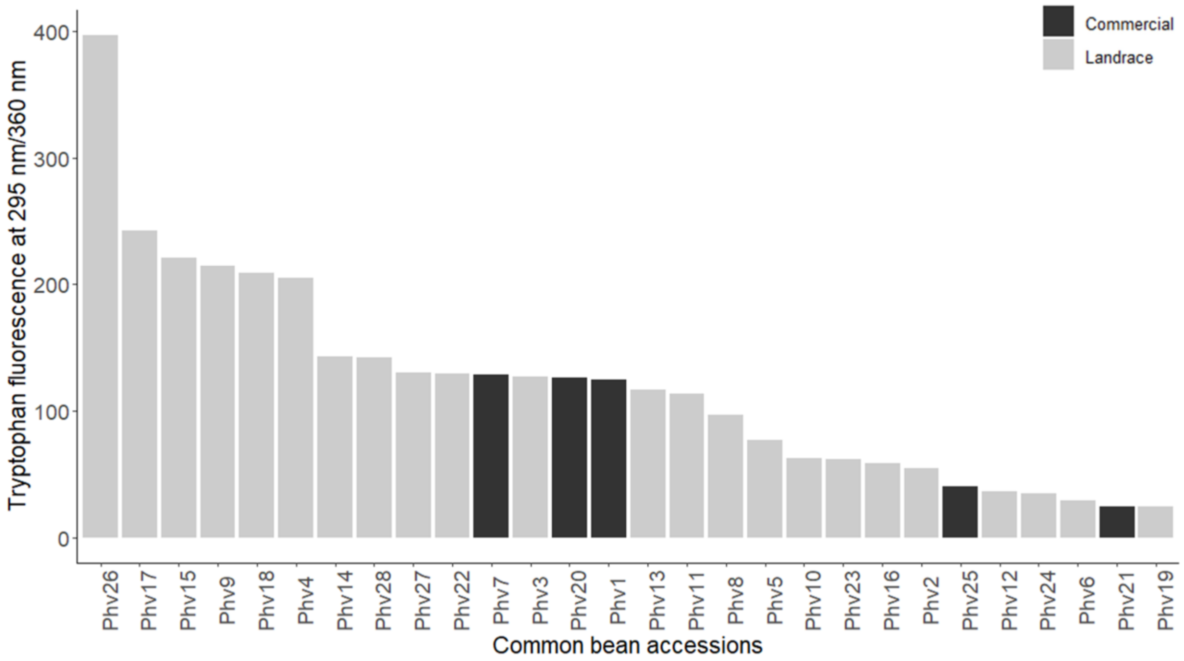

Figure S2. Content of Tryptophan (Trp) evaluated by fluorescence for 28 common bean accessions evaluated.

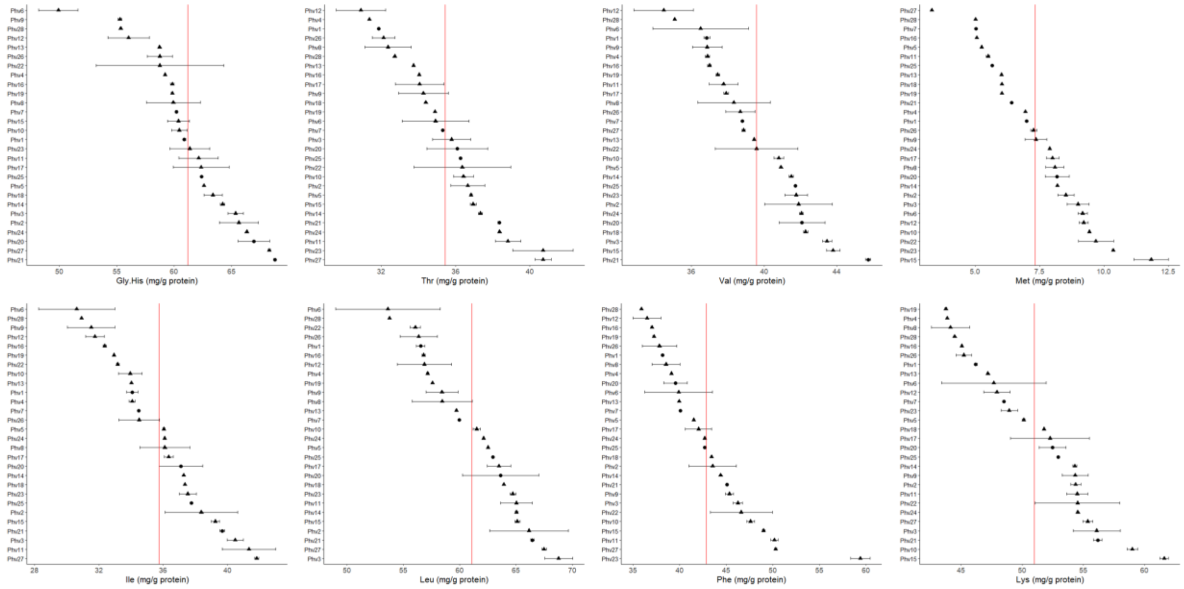

Figure S3. Mean concentration and standard deviation of essential amino acids (EAAs) for the 28 common bean accessions. Chilean landraces and commercial varieties are indicated with a triangle and a circle in the scatterplots, respectively. The red vertical line indicates the average concentration of each element in all accessions. Significant differences between each accession according to the Tukey test are shown in the Table S2.

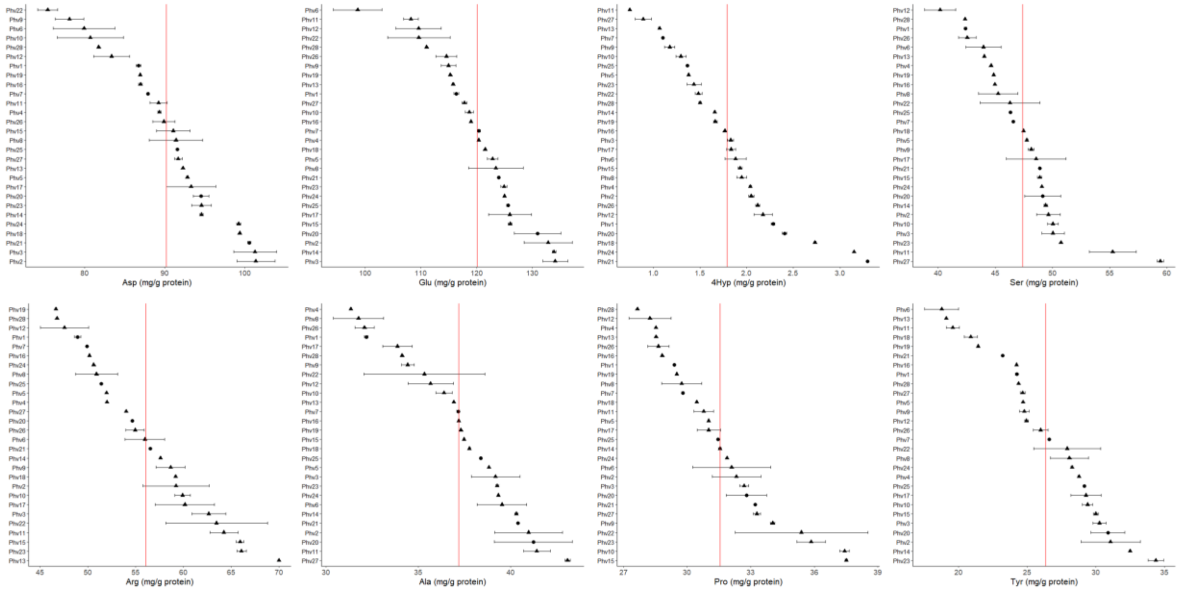

Figure S4. Mean concentration and standard deviation of non-essential amino acids (NEAAs) for the 28 common bean accessions. Chilean landraces and commercial varieties are indicated with a triangle and a circle in the scatterplots, respectively. The red vertical line indicates the average concentration of each element in all accessions. Significant differences between each accession according to the Tukey test are shown in the Table S2.

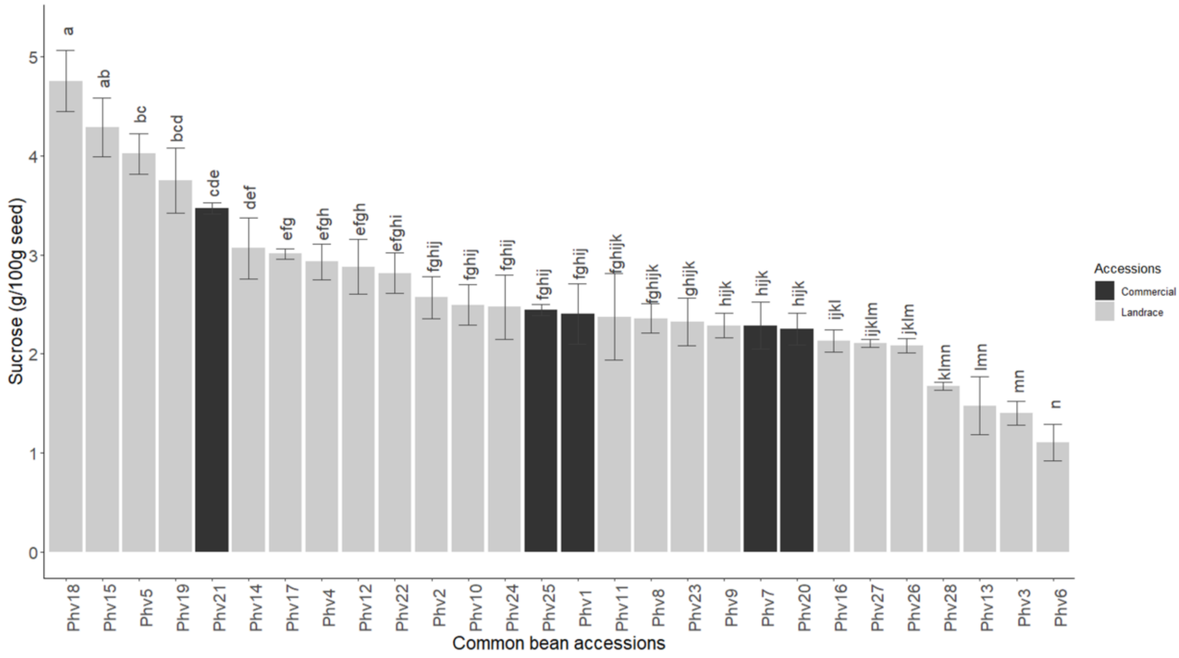

Figure S5. Content of sucrose for the 28 common bean accessions evaluated. Bars with different letters are significantly different ($p < 0.05$) according to Tukey test.

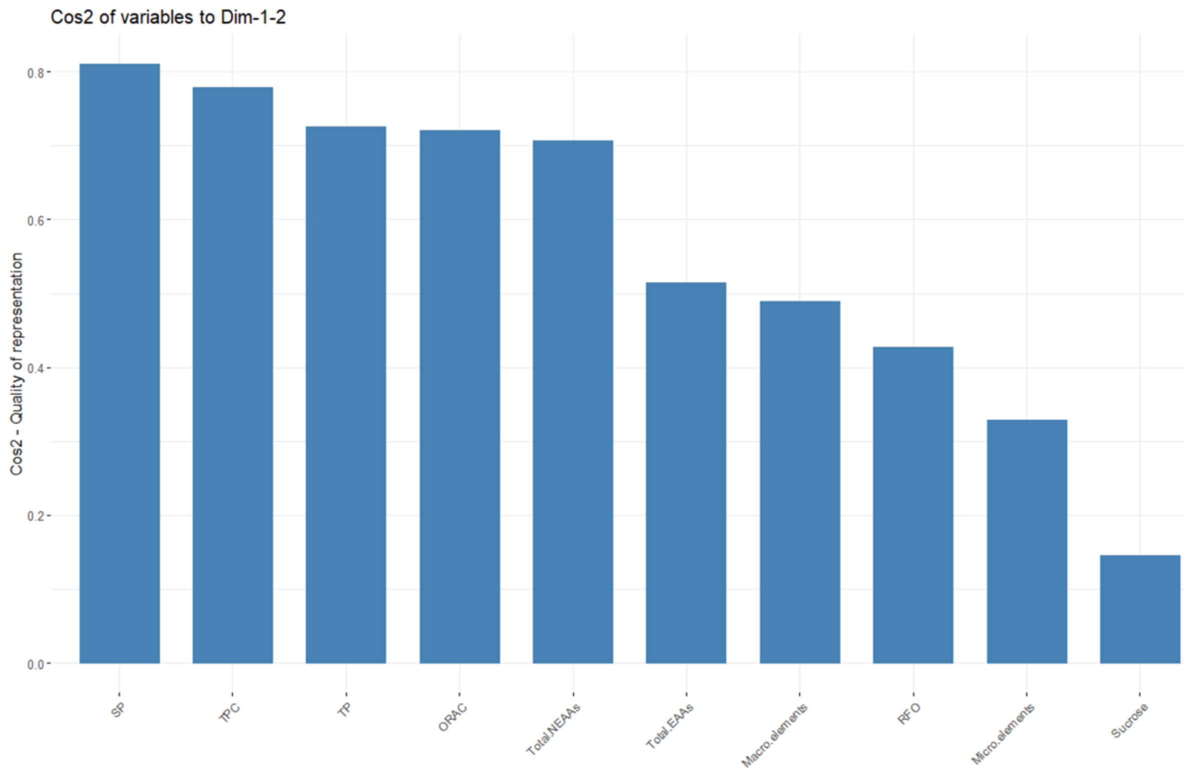

Figure S6. Loading for the variables that most contribute to the first two principal components.

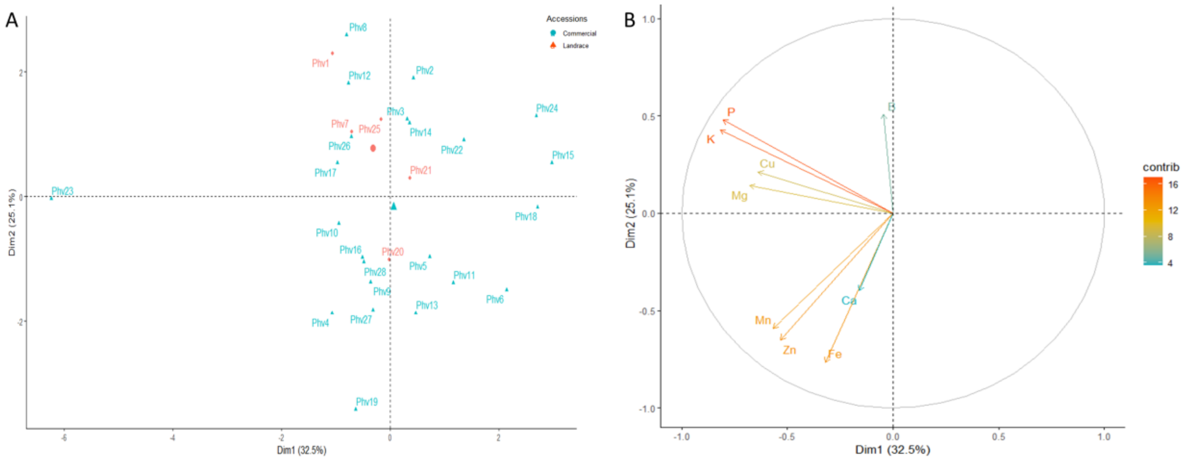

Figure S7. PCA based on minerals (micro and macro-elements).

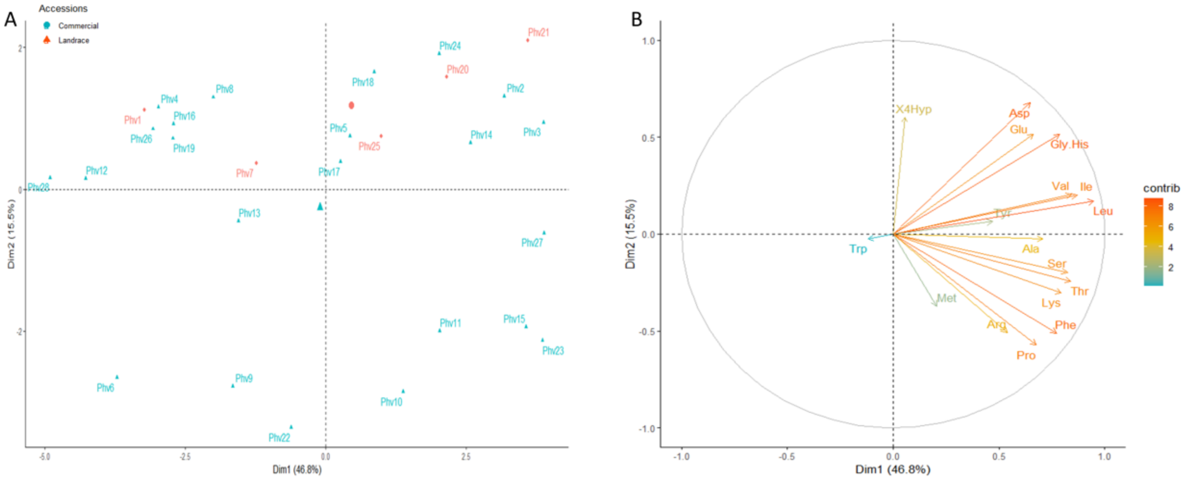

Figure S8. PCA based on amino acids (essential and non- essential).
